# Integrating Behavioral Science-Based Interventions in the Treatment of Refractory Atopic Dermatitis and Associated Behavioral Problems

**DOI:** 10.31662/jmaj.2025-0070

**Published:** 2025-11-21

**Authors:** Marei Omori, Tomoki Yaguchi, Chisato Jimbo, Kouhei Hagino, Daisuke Harama, Daichi Suzuki, Kotaro Umezawa, Fumi Ishikawa, Seiko Hirai, Kenji Toyokuni, Tatsuki Fukuie, Yukihiro Ohya, Kiwako Yamamoto-Hanada

**Affiliations:** 1 Allergy Center, National Center for Child Health and Development, Tokyo, Japan

**Keywords:** atopic dermatitis, behavioral science, problematic behaviors, tantrum

## Abstract

This report highlights the successful integration of behavioral science-based interventions in the treatment of an 8-year-old girl with atopic dermatitis (AD) and behavioral problems, including tantrums. The patient, who had a history of poorly managed AD, presented with worsened eczema and sleep disturbances, in addition to increased tantrums, scratching, and reluctance to attend school. She was admitted for inpatient treatment, which included standard AD management and multidisciplinary care, focusing on proper skin care, diet, and lifestyle changes. Despite relief of her eczema, her problematic behaviors persisted, which worsened her condition.

To address this, behavioral interventions were implemented. Anger management techniques were introduced, whereby the patient assessed her anger levels and used relaxation methods. She was taught alternative behaviors to manage the itching, such as applying topical treatments or interacting with her favorite stuffed animal. Her parents also received guidance on managing her behavior. Over her three-month hospitalization, her tantrums and problematic behaviors resolved, and her AD went into remission. Post-discharge, her eczema remained well controlled, and her behavior improved, with no recurrence of tantrums or other issues.

This case illustrates the importance of combining pharmacological treatments with behavioral interventions in managing AD and associated psychosocial challenges. Behavioral science-based approaches can play a crucial role in alleviating behavioral issues linked to AD, such as anger and scratching, which can further exacerbate the condition. In this case, behavioral therapy effectively alleviated the patient’s behavior problems and improved eczema control, underscoring the need for clinicians to be equipped to address both the physical and behavioral aspects of AD.

## Introduction

Atopic diseases can cause psychosocial problems and have a significant impact on quality of life. It has already been reported that atopic diseases are associated with problematic behavior ^(1)^. In a US cohort study, children with atopic dermatitis (AD) had greater odds of caregiver-reported internalizing and externalizing problems than did those without AD, independent of sociodemographic characteristics and atopic comorbidities. In another US report, childhood AD, a particularly persistent disease with sleep disturbances, was associated with a wide range of behavioral problems in childhood and adolescence ^(2)^. In the treatment of atopic diseases, not only medication but interventions for problematic behavior are needed. According to Ohya ^(3)^, behavioral medicine is a comprehensive approach that adapts a bio-psycho-sociological model, and it could improve the patient’s behavior in a short period by combining medical formula and behavioral therapy. In Japanese medical schools, behavioral science has only recently become a mandatory subject. However, the reality is that many physicians remain unable to implement treatments based on behavioral science in their practice.

We report on the course of inpatient treatment in children with AD with tantrums, combined with behavioral science-based interventions.

## Case Report

Case: Girl aged 8 years

Chief report: Worsening of eczema, sleep disturbances

Medical history: Unremarkable

AD developed in the patient in infancy, and she received treatment with topical corticosteroid and emollient. Owing to inadequate treatment, she had repeated remissions and exacerbations of eczema. In the spring of her eighth year, her eczema worsened, and sleep disturbances appeared. She was reluctant to go to school every day, cried loudly in public, scratched violently, and had frequent tantrums. She visited our center in the summer of the same year and was admitted for induction remission treatment for AD.

**Figure 1.**
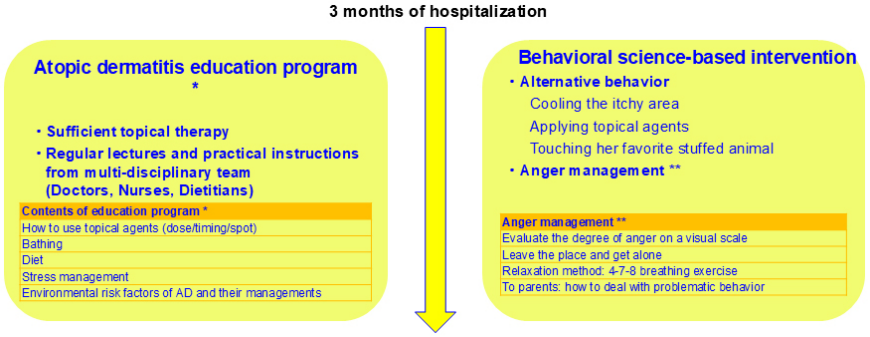
Clinical course of hospitalization.

We educated the patient about AD and provided her with standard treatment including topical corticosteroids, topical difamilast, and emollient (Figure 1). She did not receive any systemic therapy because it was not approved at that time. A multidisciplinary team, including doctors and nurses, gave her thorough topical therapy and taught her practical bathing and skin care techniques. We also explained to the patient and her parents the importance of a quality diet and a regular lifestyle and guided them in environmental modifications. After the start of inpatient treatment, her eczema was steadily relieved, but she had occasional tantrums and violent scratching, which worsened her eczema. We had to work carefully to keep her in a good mood.

The patient’s problematic behavior had to be improved to relieve her eczema. For her anger and tantrums, we based our interventions on behavioral science. First, we taught her anger management. When she felt angry, she assessed the level of anger on a visual scale, left the situation to be alone, and performed relaxation techniques with breathing exercises. Second, she performed alternative behaviors when she felt itchy. Specifically, she practiced her own methods of cooling the itchy area, applying additional topical medication, and touching her favorite stuffed animal. Third, her parents were instructed on ways to deal with problematic behavior.

During her three-month hospitalization, the patient’s tantrums and problematic behavior disappeared, and her AD went into remission. After discharge, her AD is well controlled. In addition, her tantrums have not flared up, and she has no problematic behavior. She attends school willingly.

## Discussion

To the best of our knowledge, this was the first successful report to treat both severe refractory AD and tantrums. AD cannot be fully resolved through pharmacotherapy alone; however, when integrated with non-pharmacological methods, such as evidence-based behavioral therapy, remission of AD has been attained. The patient’s problematic behavior could be attributed to itch and lack of sleep caused by severe eczema. Nevertheless, the tantrums persisted even after the itch had resolved and the patient could sleep well. Behavioral science-based interventions caused the complete disappearance of her tantrums and problematic behaviors and better control of her eczema.

AD in adolescents is associated with mental health concerns, including disruptive behavior, symptoms of anxiety and depression, and diagnoses of attention deficit/hyperactivity disorder and autism spectrum disorder ^(4)^. There is also a past report of behavioral problems being exacerbated when AD persists ^(2)^. Sleep plays a crucial role among the key mechanisms investigated thus far, with evidence indicating that it serves as a mediator in the connection between AD and psychiatric symptoms. Moreover, there is evidence supporting various other mechanisms contributing to the emergence of mental health issues in pediatric AD, such as itches, pain, comorbid conditions, and the use of systemic antihistamines.

Teaching anger management skills to patients with human immunodeficiency virus alleviated their anger status ^(5)^. Clinicians need to acquire the skills to intervene not only with medication-based therapies but also with problematic behaviors.

In summary, behavioral science-based interventions for problem behaviors are essential for disease control, and they can be synergistic with treating atopic diseases.

## Article Information

### Author Contributions

Marei Omori and Kiwako Yamamoto-Hanada established the concept of this case study. Tatsuki Fukuie and Yukihiro Ohya supervised this case. All authors followed the case in the hospital. Marei Omori wrote the first draft of the manuscript. All authors critically reviewed the manuscript and approved the final version.

### Conflicts of Interest

None

### IRB Approval Code and Name of the Institution

Not applicable. Informed consent was obtained from the patient’s parents.

## References

[1] Ma EZ, Hooper SR, Seegan PL, et al. Association of atopic dermatitis with emotional and behavioral problems in childhood. J Am Acad Dermatol. 2024;90(6):1249-52. 38320626 10.1016/j.jaad.2024.01.068 PMC11096002

[2] Manjunath J, Silverberg JI. Atopic dermatitis is associated with multiple behavioral problems in US children and adolescents. Dermatitis. 2022;33(6S):S52-60. 33840782 10.1097/DER.0000000000000749

[3] Ohya Y. Therapy for childhood atopic dermatitis－psycho-behavioral approach. Hifu Kagaku. 2004;3(suppl 4):76-80.

[4] Radtke S, Grossberg AL, Wan J. Mental health comorbidity in youth with atopic dermatitis: a narrative review of possible mechanisms. Pediatr Dermatol. 2023;40(6):977-82. 37665064 10.1111/pde.15410 PMC10863653

[5] Lotfalizadeh M, Miri S, Foroughameri G, et al. The effect of anger management skills training on anger status of the people with HIV. Perspect Psychiatr Care. 2020;56(3):605-13. 31984531 10.1111/ppc.12475

